# Learning Through Digital Stories for Safe School Environment

**DOI:** 10.3389/fpsyg.2021.738954

**Published:** 2021-09-24

**Authors:** Sahin Akdag, Zehra Altinay

**Affiliations:** Department of Computer and Instructional Technologies, Faculty of Education, Near East University, Nicosia, Cyprus

**Keywords:** special education, animation, COVID−19, digital story, safe school environment

## Abstract

This study aimed to evaluate the impact of digital stories in the learning-based themes of safe schools and to examine the perceptions of special educational needs in safe school environments. Training were carried out with informative videos created through the Distance Education and Information Technology Center (UZEBIM) for principals and teachers-in-charge, and the effectiveness of this process was evaluated through reflective opinion forms. In addition to this, an evaluation form was presented to the prospective special education teachers to obtain their opinions and evaluate the effectiveness of digital stories at safe schools. A total of 100 prospective teachers participated in the evaluation of the impact of digital stories on their learning about safe school environments. Digital stories regarding safe schools become an important source of information in creating a safe school environment more rapidly and efficiently. In line with the interviews, it was observed that the awareness of safe school environments was increased and the digital dimensions of safe school environments were internalized through digital stories. Due to the raising awareness in the COVID-19 process, it was revealed that the schools did not have sufficient safe school characteristics and all stakeholders should take preventive measures in coordination to establish a safe school environment.

## Introduction

The concept of a safe school environment has become important in education after the COVID-19. During the pandemic, the education systems have been affected due to health issues, and having a safe school environment has turned to be an essential issue in schools. On the other hand, the integration of technology into learning activities plays a great role to increase the awareness of the concept of safety in the practice (McBrayer et al., 2020; Packy-Green et al., 2020). Huang et al. give insights on open educational resources during school closures (Huang et al., 2020). This is an example of how the integration of technology can support the school systems. The quality of education relies on safe schools to foster learning activities (Guillemard, 2020).

A safe school environment is not limited to stakeholders who know how to protect their rights on various issues. It also includes the process of developing the school positively to create a strong commitment to the school with students, teachers, staff, families, and the school environment. No matter how complicated or multidimensional the concept of safe school is, it can be argued that if schools are aimed to be environments where students can meet their personal, social, and academic needs, the issue of safe school requires more attention due to various reasons emerging expectedly or unexpectedly from time to time.

The Safe Schools Declaration was issued in 2015 to reduce various issues causing conflict in education. Building capacity for teachers and improving school environments through subject-based training in safe schools turned to be essential. In addition, UNESCO emphasizes the measuring of the progress of Sustainable Development Goal 4 on Education. This goal relies on safe and non-violent learning environments for all children and adolescents. In this respect, achieving this target requires researchers to consider the theme of “provide safe, non-violent, inclusive, and effective learning environments for all” for the quality of education and sustainability through social justice. As UNESCO underlines the intensified need for safe, non-violent, inclusive, and effective learning environments, having a comprehensive look at safe school frameworks and the integration of digital technologies on learning play an important role in the establishment of safe school environments.

Schools become social agents to integrate digital transformation to enhance learning and teaching. In this respect, special education schools require the integration of principles of inclusive education. Therefore, in line with the principle of sustainable growth, the safe school policy within the aspect of inclusive education is essential. The study of Terrell et al. points out the importance of a comprehensive framework of safe and supportive schools (Terrell et al., 2020). In this respect, some essential issues such as physical safety and security and also emotional safety, physical environment, engagement, and norms and policies to be applied are to be considered.

Some individuals may differ significantly from their peers in terms of both their characteristics and their educational competence due to their different conditions. Depending on their age, gender, and social and cultural differences, individuals may also have some obstacles in terms of inability preventing to fulfill their roles adequately. As a result, some individuals may need to be engaged in special education. Special education can be defined as all of the educational services that are provided to students, which differ greatly from the average characteristics of students and are aimed to maximize the probability of individuals living without being dependent on anyone (Sari, 2012). Special education is a kind of education provided to individuals who are different from the majority and necessitate the application of special education programs, (i) ensuring that gifted individuals receive an education convenient for their gifts and that they make maximum use of these gifts, (ii) preventing the inadequacies of individuals from turning into disabilities, and (iii) supporting people with disabilities so that they can become self-sufficient, can integrate with the society, can behave independently, and can become productive individuals (Çankaya et al., 2014).

Stephens listed the factors affecting school security as follows: the personal behavior of all students and employees, the social and physical environment of the school, the behavior of school employees and students, and the economic state of the society that maintains its life around the school (Dönmez and Özer, 2010). The development of technology and its use in social institutions has caused a positive change in daily life. The use and dissemination of information and technology in special educational institutions along with individual differences is of great importance in terms of creating an effective learning environment. The use of computer software and technology that will facilitate the learning of individuals with special education will be a major factor both in responding to the needs of the individuals and in solving the problems they face in their daily lives. Again, using the technologies related to safety in schools can provide benefits in terms of reducing, monitoring, and intervening in a very short time to the safety problems that may occur in schools (Dönmez, 2001).

It is even more important that institutions with individuals requiring special educational needs have been turned into safe schools. Undoubtedly, in terms of school safety, students are primarily in need of protection in every aspect. On the other hand, the events show that administrators, teachers, and other employees are not safe enough (Hughes-Roberts et al., 2020). In this context, different theoretical perspectives on a safe school environment and dimensions of a safe school environment should be comprehensively addressed.

As described in detail in the above statements, the concept of a safe school environment is complex, and when it is handled with traditional education methods, it may not reflect the efficient processes in terms of time and economy. Therefore, the digital story method is used in this study to analyze and internalize these important concepts.

Digital games, which can be regarded as forms of digital stories in the current status, have also become important in education. They are now regarded as part of the tools for education activities in facilitating learning. Giving a chance for engaging learning and participation also makes an improvement in teaching capacity (Zhonggen, 2019). Games are beneficial for learners, especially for adults on their well-being (Chang et al., 2008) in terms of their emotional moods. The studies show that there are benefits and positive effects of training through video games on developing cognitive and emotional skills. The study of Zhonggen gives insight on trends in games and learning that games play an important role in improving learning effectiveness and in enhancing the learning experience (Zhonggen, 2019). In this respect, educational technologies, such as games and mobile applications, facilitate improvement in academic achievements and give a change to individuals to participate in learning activities. Thus, most games have turned into an effective tool in improving teaching-learning facilities. Games make learners have higher motivation in learning and improve willingness in learning actively (Chang et al., 2008).

With the emergence of the digital game approach, the physical-based traditional game approach is replaced by digitally produced game environments. The digital game is the new communication environment standing out as an individual communication environment that includes digitality, interactivity, virtuality, variability, and modularity and adds these features to the act of playing games (Hughes-Roberts et al., 2020).

When compared to traditional games, the phenomenon of digital games indicates a structure that develops outside of certain standards and is systematic within itself but generally quite dynamic. What makes the phenomenon most prominent in form and content is that it allows player-oriented communication. In digital games, the result is determined by the actions of the player. In the sense that the actor, other players, and events affect each other, interactivity is the primary concept that determines the game phenomenon in digital form (Flynn et al., 2019).

The interactive communication opportunities offered by digital games to individuals create a brand-new socialization area for them. Personal profiles defined through the game characters or avatars make the player the leading character of the story with a very realistic story that immerses the individual and increases the permanence (Lee, 2019).

In this regard, digital stories in games are an effective learning and teaching instrument for students and teachers. For students, the digital story process is open to learning different skills. A digital story that personalizes the learning process contributes to the development of the skills of students such as research, writing, organization, technology, presentation, interview, problem-solving, and evaluation (Altinkurt and Yilmaz, 2011). It is a teaching instrument that offers teachers the opportunity to integrate technology into their lessons.

In digital storytelling (Robin, 2008), which is a student-centered practice, teachers give ideas to the students by providing guidance (Vinogradova et al., 2011).

In education, the digital storytelling and writing process is a powerful teaching instrument (Wawro, 2012). Through digital storytelling, students can learn how to write good stories, integrate text and art, and use technology in a creative way (Miller, 2010). In addition, if students pay attention to the writing process, by embracing their stories, they can participate more effectively in the process of creating digital stories and can make the digital story more effective and successful with a good scenario (Xu et al., 2011). Digital storytelling offers many opportunities for teachers and students to listen to the lesson.

The research conducted in this context is important in terms of evaluating the participant views on school safety in terms of contributions of digital stories. It is thought that revealing the opinions of the participants on this subject will benefit the literature. This study is important to reveal the need for an environmentally sensitive, production-encouraging approach to school safety at present and in the future.

## Materials and Methods

This study relies on qualitative and quantitative research based in a descriptive way. The research has two phases in its nature. In the first phase, the focus of the research aims to increase the awareness of safe schools and to internalize safe school dimensions in special education institutions. In line with the general aim, the research questions of the study are as follows:

1. What are the views of school principals and teachers on special education institutions in terms of safe school?

2. What are the views of students from the department of special education on the digital stories in terms of various variables?

### Research Steps on Phase 1 and Phase 2

#### Phase 1

To achieve the aims of this study, training was provided to the principals and responsible teachers with informative videos and seminars created through the Distance Education and Information Technology Center (UZEBIM). In this phase of the research, the study group consists of the principals and teachers in six special education centers in the Turkish Republic of Northern Cyprus in 2018–2019. In the second phase, the aim of the research was to evaluate the stories in terms of their scenarios as digital stories were created based on the Plotagon animation program and video creation based on Camtasia Studio 2019.

In addition, voices were created based on the artificial intelligence voice applications from the web and also the voice of the researcher. Scenarios were created based on the content of the seminars of experts. Seminars were conducted with students through digital stories. In this respect, the digital storytelling method is also used to enable participants to experience active participation in the teaching process. As one of the aims of this research is to raise awareness of the safe school concept in learning environments, videos were created by experts on the safe school environment, and also digital stories on safe school issues were created for students to increase their awareness in learning at safe schools.

In the research, the following themes are considered as seminars and also for digital stories.

#### Phase 2

The data were collected through a semi-structured interview form. Qualitative research has been more concerned in terms of the products and outputs as meanings are important in qualitative research (Altinkurt and Yilmaz, 2011). Semi-structured interviews are frequently preferred by researchers because of their certain level of standard and flexibility, eliminating the restrictions in tests and surveys based on writing and filling and helping to gain in-depth knowledge on a particular subject (Karataş, 2015). Regarding the validity and reliability issues of the study, the data collection tool included interview forms, i.e., draft statements that were given to each of the institutions as a pre-application after expert opinions were obtained and necessary arrangements were made. After analyzing the results, the final version of the data collection tool was developed. The data collection tool was prepared as a semi-structured interview form consisting of six basic themes. After the obtained data were pre-applied and the data collection tool was finalized, interviews were carried out with the administrators working in special education centers.

The questions in the interview form were processed in terms of four subthemes (i.e., school, classrooms-workshops, playing-social field, and technological infrastructure). The opinions of the participants were obtained as follows: In “Opinions on Physical and Architectural Safety” theme, there are three subthemes (i.e., student-parent-teacher interaction, special security, and violence-bullying among students); in “Opinions on Evaluation of School Climate and Culture” theme, there are three subthemes (i.e., cleaning and environmental risks, contagious diseases and rehabilitation, and health services); and in “Opinions on Health Safety” theme, there are “Opinions on Emergencies and Crisis Management,” “Opinions on Transportation Opportunities,” and “Opinions on Technological Devices and Competence.”

**Figure 1:**
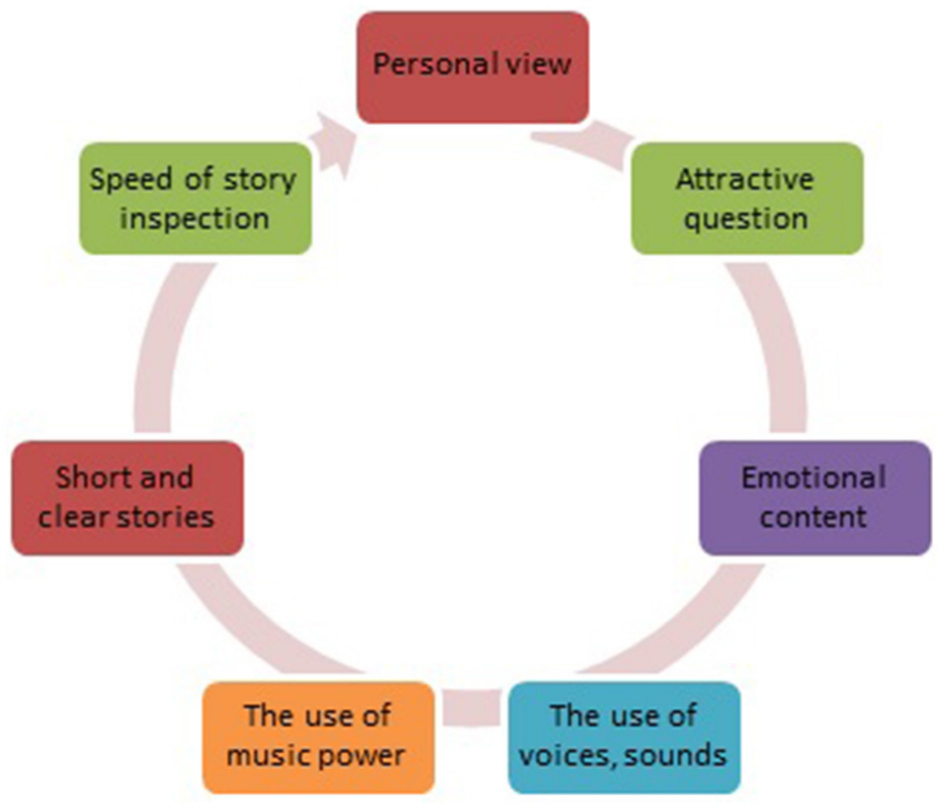
The type of digital stories used is to teach and inform.

In the second phase, digital stories on safe schools were conducted as a course to special education students as a subject of teaching, and their learning and motivation levels were assessed. As Figure 1 shows there are different types of digital stories designed to teach and inform. After sharing stories in the course, a scale was applied to get reflections of students on digital stories related to the safe school environment. In addition to this, a scale, which was adapted from Ozcan on the Digital Story Evaluation Scale, was conducted on the prospective special education teachers. In addition to a scale, three reflection questions were asked to students to evaluate the learning and motivation factors of digital stories in a safe school environment (Özcan et al., 2016).

While digital stories were being prepared, the following criteria were taken into consideration (Lambert, 2003).

For the effectiveness of the video-based seminars, reflective opinion forms were given to the principals and teachers. On the other hand, the impact of digital stories was also evaluated by using the reflective opinion form to set motivation and make a comparison between traditional learning and digital story-based learning. Digital stories were created based on seminars, and in the form, the following criteria were asked to four experts in educational technology and four experts in language to evaluate the availability of created digital stories to be used in learning.

In this study, it was aimed to increase the awareness of safe school environments and to internalize safe school dimensions in special education institutions through digital stories. Accordingly, as a result of evaluations made with school administrators, the special education institutions were also examined within the scope of the safe school concept and have been determined that there are inadequacies in physical and architectural safety, transportation facilities and technological infrastructure, and deficiencies in terms of school climate and culture, health safety, and emergency and crisis management. In line with the interviews, it has been found out that schools do not have a sufficient level of school safety and that the ministry, school management, and all stakeholders should take preventive measures in coordination among themselves to establish a safe school environment. In addition, it has been understood that the use and dissemination of technology in special educational institutions with individual differences is of great importance in terms of creating an effective learning environment. It is thought that the use of technology is an essential part of education both in responding to the needs of the individual and in solving the problems in their daily lives.

## Results

Opinions of the participants were conveyed based on confidentiality and were coded anonymously. Accordingly, to identify the concept of a safe school environment for the participants, coding was performed as “G,” and each participant was given a number such as G1, G2, G3, G4, G5, and G6 beside their code. In this part of the research, the questions in the interview form are grouped by themes, and the findings are presented.

### Opinions on Physical and Architectural Safety

There are four subthemes (i.e., school, classrooms-workshops, playing-social field, and technological infrastructure) in the “Opinions on Physical and Architectural Security” theme. The opinions that emerged as a result of the analysis of the answers received in line with the “Opinions on Physical and Architectural Security” theme are as follows:

#### Opinions on the Physical and Architectural Location Subtheme of the School

The opinions of the administrators participating in the research about the physical and architectural location of the school are shown in Table 1.

**Table 1:** Views on the physical and architectural location of the school.

**Codes**	***f***	**%**
Physical location is convenient	3	25
Architectural location is convenient	3	25
Physical location is convenient, architectural location is not convenient	1	8
Physical location is convenient	2	17
Architectural location is not convenient	3	25

As shown in Table 1, one administrator participating in the study stated that his physical location was appropriate, but the architectural location was not appropriate (G1). Two administrators stated that the physical and architectural location is not suitable (G3 and G5).

The opinions of the administrators reflecting this situation are given below.

“Our school is suitable as a physical location, but we are having difficulties because it is two-story as an architectural location” (G1).

“Our school is not physically and architecturally suitable. It is not equipped to meet the needs of individuals with special needs. We are having a lot of troubles” (G3).

Stating that the institution is physically and architecturally appropriate (G2, G4, and G6) argues that the technological equipment is not sufficient.

The opinions of the administrators reflecting this situation are given below.

“It is a great advantage for our school to be on a dead-end street as a physical location. We have an architecturally appropriate building. Suitable for the proper use of each individual. However, technological equipment is not enough” (G2).

The Accessibility Guide prepared by the Izmir Chamber of Architects as a result of detailed research describes the disabled individuals as follows: “people who suffer from disabilities due to the lack of suitable facilities in their use of buildings designed for general needs due to their physical deficiencies.” Especially, the institutions where individuals with special needs are educated should have features that meet the needs of all individuals. In this regard, huge responsibility falls on individuals and especially state administrators.

#### Opinions on Classrooms and Workshops, Playing-Social Field, and Technological Infrastructure Subthemes

As shown in Table 2, two of the administrators participating in the research stated that their classrooms and workshops were satisfactory (G1 and G3).

**Table 2:** Opinions on classrooms and workshops, playing-social field, and technological infrastructure subthemes.

	***f***	**%**
**Opinions on classrooms and workshops codes**		
Satisfactory	2	33
Not satisfactory	4	67
**Opinions on playing-social field codes**		
Satisfactory	4	67
Not satisfactory	2	33
**Opinions on technological infrastructure codes**		
Not satisfactory	6	100

The opinions of the administrators reflecting this situation are given below.

“Our classrooms and workshops are sufficient for our students. It is sufficient because interior designers are prepared in line with the needs of our students” (G3).

Four of the administrators participating in the research stated that their classrooms and workshops are not satisfactory (G2, G4, G5, and G6).

The opinions of the administrators reflecting this situation are given below.

“Our classrooms and workshops are not equipped satisfactorily. Especially, the workshops are inadequate in terms of both equipment and personnel” (G2).

As shown in Table 2, four of the administrators participating in the research claimed that their playing-social areas are sufficient (G1, G2, G4, and G5).

The opinions of the administrators reflecting this situation are given below.

“Our playing-social fields are sufficient. We have received support from some organizations as sponsors” (G1).

“Our playground is convenient and safe. We also have a park as a social field and a hall for our events” (G2).

Two of the administrators participating in the research stated that their playing and social areas are not sufficient (G1, G2, G4, and G5).

The opinions of the administrators reflecting this situation are given below.

“Our playing-social fields are sufficient. We have received support from some organizations as sponsors” (G1).

“Our playground is convenient and safe. We also have a park as a social field and a hall for our events” (G2).

As shown in Table 2, all of the administrators participating in the research stated that their technological infrastructure is inadequate (G1, G2, G3, G4, G5, and G6).

The opinions of the administrators reflecting this situation are given below.

“Our technological infrastructure is inadequate. It is very important to have a technological infrastructure that our individuals can use according to all disability groups” (G4).

“We just have computers. However, it is not suitable for all types of disabilities. Therefore, it does not meet the needs of the vast majority” (G2).

## Discussion and Conclusion

In this study, it was aimed to increase the awareness of safe school environments and to internalize safe school dimensions in special education institutions through digital stories. Accordingly, as a result of evaluations made in cooperation with school administrators, the special education institutions were assessed within the scope of the safe school concept. In line with the interviews, it has been displayed that schools do not have a sufficient level of school safety and that the ministry, school management, and all stakeholders should take preventive measures in coordination among themselves to establish a safe school. In addition, it has been understood that the use and dissemination of technology in special educational institutions with individual differences is of great importance in terms of creating an effective learning environment. It is thought that the use of technology is an essential part of education both in responding to the needs of the individual and in solving the problems in their daily lives.

In this context, it is necessary to give the necessary training to first prepare the technological infrastructure and then to make the best use of technology. As stated in the “Materials and methods” section, the digital storytelling method can be used as a tool or a mediator in this process of increasing the awareness of safe schools and internalizing the safe school concept in special education institutions. Challenging classwork, technology-driven discourses, and innovative assignments may motivate the students to have positive communication in the classroom (Zahid et al., 2021). The benefits of digital storytelling, on the other hand, can be emphasized as enabling people to transfer information to each other, giving them individuality, increasing the use of technology, and improving critical thinking. As for the digital story types, mostly for educational purposes, the stories prepared for information about a topic are used. In this respect, in this study, according to the aim that digital storytelling mediating to increase the awareness of safe schools and to internalize safe school dimensions in special education institutions, it has been determined that there are inadequacies in physical and architectural safety, transportation facilities and technological infrastructure, and deficiencies in terms of school climate and culture, health safety, and emergency and crisis management. In line with the interviews, it has been displayed that schools do not have a sufficient level of school safety and that the ministry, school management, and all stakeholders should take preventive measures in coordination among themselves to establish a safe school. In addition, it can be stated that the use and dissemination of technology in special educational institutions with individual differences is of great importance in terms of creating an effective learning environment.

Digital stories can be utilized as both teaching and learning tools. Rieger et al. discuss the importance of digital storytelling by underlying the merits of the arts-based research method (Rieger et al., 2018). This method increases the engagement of the participant through multimedia materials to share experiences. Stories are the pedagogical tools to foster learning and motivation. It is noted that they have positive impacts on improving the reading, writing, and creative skills of the students.

## Data Availability Statement

The original contributions presented in the study are included in the article/supplementary material, further inquiries can be directed to the corresponding author/s.

## Ethics Statement

The studies involving human participants were reviewed and approved by Near East University Ethical Committee. The patients/participants provided their written informed consent to participate in this study.

## Author Contributions

SA: contributed to conception and design of the study and wrote sections of the manuscript. ZA: wrote the first draft of the manuscript. Both authors contributed to manuscript revision, read, and approved the submitted version.

## Conflict of Interest

The authors declare that the research was conducted in the absence of any commercial or financial relationships that could be construed as a potential conflict of interest.

## Publisher's Note

All claims expressed in this article are solely those of the authors and do not necessarily represent those of their affiliated organizations, or those of the publisher, the editors and the reviewers. Any product that may be evaluated in this article, or claim that may be made by its manufacturer, is not guaranteed or endorsed by the publisher.
